# Comparison Between Inertial Sensor and Video-Based Detection of Spatiotemporal Limb Movement Parameters During Equine Swimming

**DOI:** 10.3390/s26092743

**Published:** 2026-04-28

**Authors:** Frederic Marin, Chloé Giraudet, Pauline Gaulmin, Claire Moiroud, Emeline De Azevedo, Chloé Hatrisse, Khalil Ben Mansour, Pauline Martin, Fabrice Audigie, Henry Chateau

**Affiliations:** 1 Department of Movement Science for Prevention and Rehabilitation, Institute of Human Movement Science and Health, Chemnitz University of Technology, D-09111 Chemnitz, Germany; 2 Laboratoire de BioMécanique et BioIngénierie (UMR CNRS 7338), Université de Technologie de Compiègne (UTC), F-60200 Compiègne, France; 3 Unit Diseases of the Athlete Horse: Pathophysiology, Prevention, Management (ACAP3), Ecole Nationale Vétérinaire d’Alfort, F-14430 Goustranville, Francehenry.chateau@vet-alfort.fr (H.C.); 4 Laboratoire de Biomécanique et Mécanique des Chocs (LBMC) UMR_T 9406, Université Claude Bernard Lyon 1, F-69675 Bron Cedex, France; 5 LIM France, 24300 Nontron, France

**Keywords:** motion analysis, biomechanics, horse, wearable sensors, inertial measurement units, swimming, spatiotemporal parameters

## Abstract

Equine swimming is increasingly used for injury prevention and rehabilitation, but objective analysis of movement during swimming remains limited compared to land-based locomotion. Spatiotemporal parameters are essential for evaluating therapeutic outcomes, yet capturing these parameters is technically challenging due to difficulties in observing limb motion in water. Inertial sensors, already widely applied in equine science, offer a promising solution for measuring swimming kinematics objectively. The objective of this study was to evaluate the reliability of inertial sensors placed on equine distal limbs in detecting key spatiotemporal events during swimming by comparing it with video-based detection made by veterinarians. For the duration of the hindlimb swimming cycle, 24 data values were analysed and showed an “excellent” agreement, with an intraclass correlation coefficient = 0.96, 95% CI: 0.904–0.983, and Bland–Altmann analysis showed an upper limit of agreement of 50 ms (95% CI: 70 ms, 30 ms) and lower one of −60 ms (95% CI: −40 ms, −80 ms). The estimates of the “swimming” duty factor of the hindlimb (n = 24) demonstrated “moderate” to “excellent” with intraclass correlation of 0.82 (95% CI: 0.625–0.920) and limits of agreement of 4.39% (95% CI: 6.21%, 2.53%) and −5.28% (95% CI: −3.42%, −7.14%). The results of the forelimb were mixed, suggesting that the cycle duration and “swimming” duty factor parameters determined for this limb should be used with caution. Overall, the findings confirm that inertial sensors, particularly on the hindlimbs, provide reliable spatiotemporal measurements and are well suited for studying equine swimming.

## 1. Introduction

Equine swimming in a controlled pool environment has been proposed as a potential approach for both rehabilitation and strength training [1]. Subsequently, the trend of practicing swimming sessions became popular [2], although the evidence of benefits is still being studied. Horses are naturally able to swim [3]; however, their interlimb coordination during swimming is dependent on the individual horse and differs from terrestrial locomotion [4]. In the water, each limb undergoes a propulsion motion followed by a return motion [4]. During the propulsion motion, the limb moves from a cranial to caudal position, generating forward propulsive force [5]. Conversely, the return motion involves reversing the movement to reposition the limb to its cranial position while minimizing drag force through flexion of the distal limb joints [6]. Didactic illustrations of the propulsion and return motion of the horse can be found in Gaulmin et al. [4].

The timing descriptions of these extreme limb positions, also known as spatiotemporal events, are very useful for describing interlimb coordination and rehabilitation outcomes [7,8]. In the case of a horse’s swimming, limited data are available because determining spatiotemporal events through visual observation is constrained in an aquatic environment [9,10]. Furthermore, as horses may exhibit several types of swimming strategies that can be specific to an individual horse and even vary, it is necessary to have a reliable measurement of the spatiotemporal parameters of swimming. These parameters include the timing of the propulsion and return phases, which allow the duration of the swimming cycle to be determined, and the “swimming duty factor”, which documents the swimming strategy chosen by the horse according to Gaulmin et al. [4]. The use of inertial sensors as on-body sensors presents an alternative motion capture technique due to their pervasiveness and versatility [11]. Despite the plethora of studies conducted on equine terrestrial locomotion that have demonstrated the efficacy of inertial sensors in yielding reliable and reproducible spatiotemporal parameters [12,13], there remains a lack of standardized methodologies and established protocols for their practical implementation in the field of equine swimming research. 

Due to this specific context, the aim of the study is to analyse the reliability of detecting spatiotemporal events using inertial sensors during horse swimming sessions in a pool.

## 2. Materials and Methods

### 2.1. Data Collection

**Figure 1:**
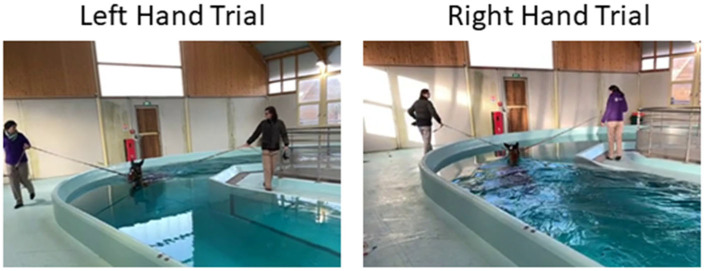
Swimming sessions with right- and left-hand trials in the U-shape pool of the equine clinic (CIRALE).

Six horses were involved for this study. They underwent a three-month training program supervised by a veterinary team at the equine clinic (CIRALE) of the National Veterinary College of Alfort (Maisons-Alfort, France). During the first month, the horses followed a conventional training programme with land-based exercises. In the last two months, however, swimming sessions were incorporated into their training routine. Each swimming session consisted of right- and left-hand trials (Figure 1), involving entering and exiting the 50 m long, 3 m deep U-shaped pool. The horses were permitted to swim at their own pace in order to prevent any fear-induced behavioural reactions. In week 8 or 5, inertial sensors are fitted to the horse, and six GoPro cameras are placed in the straight section of the pool to enable data collection. The study protocol was approved by the Clinical Research Ethics Committee of the National Veterinary College of Alfort (protocol code: 2022-09-19; date of approval: 14 November 2022).

### 2.2. Post Processing

Spatiotemporal events during horse swimming in a pool were identified using two methods.

**Figure 2:**
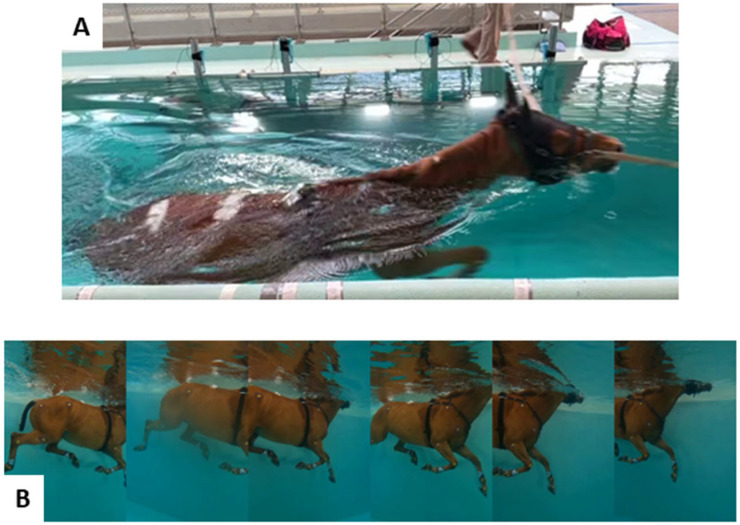
(**A**) Horse passing in front of the GoPro cameras underwater. (**B**) Video footage from the six cameras.

The first method involved underwater optical spatiotemporal event detection, performed by three experienced veterinarians, and proceeded as follows: Six GoPro cameras (GoPro Hero 8 Black, GoPro, San Mateo, CA, USA) were used in this study, with a 120-frame-per-second rate and a resolution of 2.7 K. These cameras were positioned in the straight section of the pool (Figure 2A), and their placement enabled a field-of-view of approximately 2.6 m in length and 3.0 m in height [9]. This ensures visibility of a minimum of a complete swimming cycle for each of the forelimbs and hindlimbs. Camera calibration was performed with the Camera Calibrator App of Matlab R2025b (The MathWorks, Inc., Natick, MA, USA) in order to correct optical distortion due to the wide-angle mode of the GoPro camera. A mosaic montage, incorporating footage from six GoPro videos, was achieved (see Figure 2B) [9].

Based on the mosaic montage (Figure 2B), veterinarians determined the frames marking the beginning of the propulsion (*P*1) and return (*R*1) phases of cycle 1, as well as the beginning of the propulsion (*P*2) of cycle 2 for the right forelimb (*RF*), left forelimb (*LF*), right hindlimb (*RH*), and left hindlimb (*LH*), during both right-hand trials (RTs) and left-hand trials (LTs) [4]. The timings of these events are mean values obtained by the three veterinarians. For each horse, 24 spatiotemporal events were determined and noted ${\left(t_{event}\right)}_{side,limb}^{trial}$ with *event* ∈ {*P*1,*R*1,*P*2}, Trail ∈ {*RT*,*LT*}, and side,limb ∈ {*RF*,*LF*,*RH*,*LH*} (Figure 2). 

**Figure 3:**
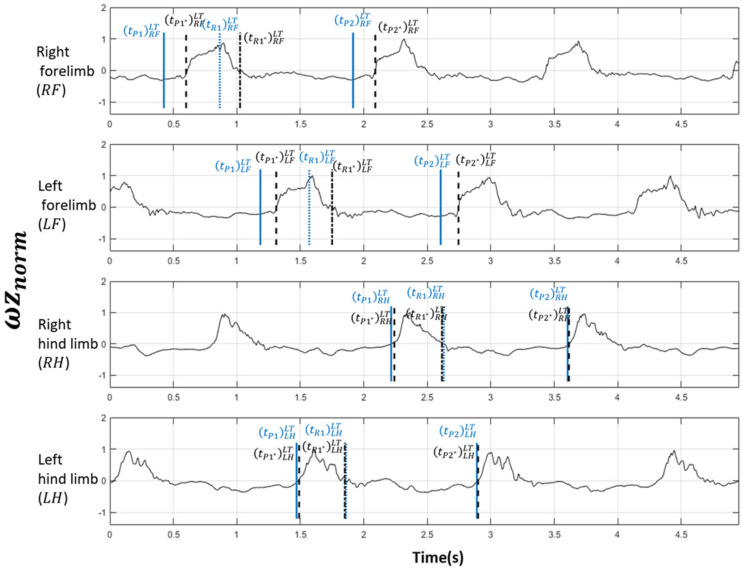
Representative results of ${\omega z}_{norm}\left(t\right)$ (Horse #5 left-hand trial). Blue lines are the timing of the spatiotemporal events determined by the visual observations of the veterinarians. Black dotted lines are the timing deduced from inertial sensors.

The second method used inertial sensors to determine an equivalent three events, denoted *P*1*, *R*1* and *P*2*. Inertial sensors (Blue Trident, Vicon Motion Systems Ltd., Oxford, UK) were attached on the lateral side of the mid-metapodium (cannon bone) region of each limb [14]. A sensor-to-segment calibration was performed [15], with the Inertial Measurement Unit (IMU) of the distal limb being manually positioned by a veterinarian in order to ensure the alignment of the Y-axis of the IMU with the transverse axis to the cannon bone [14]. The four inertial sensors were synchronized with each other and paired with a reference video using a dedicated app (Capture.U 1.4, Vicon Motion Systems Ltd., Oxford, UK). Time synchronization with the GoPro camera was achieved through a light flash at the beginning of each trial [9]. In the context of the swimming session, the rotational velocity of the inertial sensors around the z-axis was used as a tracker of the distal limb rotation, with a sampling frequency of 200 Hz [14]. The signal was normalized, yet the implementation of a filter proved unnecessary, as the motions of the limbs underwater were deemed to be sufficiently smooth. The rotation velocity is normalized by dividing this signal by the maximum absolute value of the signal; this results in a value within the range [−1, 1]. This signal was denoted ${\omega z}_{norm}\left(t\right)$, with t representing time. It was observed that the metacarpus region exhibited a propulsion movement when ${\omega z}_{norm}\left(t\right)>0$, and return movement when ${\omega z}_{norm}\left(t\right)<0$. The timing $t_{{P1}^{*}}$ of beginning of the propulsion movement of the metacarpus region of the cycle 1 was identified when ${\omega z}_{norm}\left(t_{{P1}^{*}}\right)=0$ and ${\omega z}_{norm}\left(t\to t_{{P1}^{*}}^{+}\right)$ >0 and ${\omega z}_{norm}\left(t\to t_{{P1}^{*}}^{-}\right)$ <0 with $t\to t_{{P1}^{*}}^{+}$, which means that the variable t approaches the value $t_{{P1}^{*}}$ whilst remaining greater than $t_{{P1}^{*}}$, and $t\to t_{{P1}^{*}}^{-}$, which means that the variable t approaches the value $t_{{P1}^{*}}$ whilst remaining lower than $t_{{P1}^{*}}$. The same process was repeated to identify $t_{{P2}^{*}}$ of beginning of the propulsion movement of the cannon of cycle 2. The timing of beginning of the return movement $t_{{R1}^{*}}$ of the cannon of cycle 1 was identified when ${\omega z}_{norm}\left(t_{{R1}^{*}}\right)=0$ and ${\omega z}_{norm}\left({t\to t}_{{R1}^{*}}^{+}\right)$ <0 and ${\omega z}_{norm}\left({t\to t}_{{R1}^{*}}^{-}\right)$ >0. For each horse, 24 spatiotemporal events were identified using the inertial sensor and noted ${\left(t_{{event}^{*}}\right)}_{side,limb}^{trial}$ with ${event}^{*}\in \left\{{P1}^{*},{R1}^{*},{P2}^{*}\right\}$, $trial\in \left\{RT,\;LT\right\}$, and $side,limb\in \left\{RF,LF,RH,\;LH\right\}$ (Figure 3).

Calculation of time difference ${\left(\Delta t_{event}\right)}_{side,limb}^{trial}\;$ (Equation (1)) between events identified by the veterinarians ${\left(t_{event}\right)}_{side,limb}^{trial}$ and deduced by inertial sensor ${\left(t_{{event}^{*}}\right)}_{side,limb}^{trial}$.

(1) $${\left(\Delta t_{event}\right)}_{side,limb}^{trial}={\left(t_{event}\right)}_{side,limb}^{trial}-{\left(t_{{event}^{*}}\right)}_{side,limb}^{trial}$$

The swimming cycle durations derived from the veterinarian’s data, denoted as ${\left(CycleDuration\right)}_{side,limb}^{trial}$ (Equation (2)), and from the inertial sensor data, denoted as ${\left({CycleDuration}^{*}\right)}_{side,limb}^{trial}$ (Equation (3)), were also compared.

(2) $${\left(CycleDuration\right)}_{side,limb}^{trial}={\left(t_{P2}\right)}_{side,limb}^{trial}-{\left(t_{P1}\right)}_{side,limb}^{trial}$$

(3) $${\left({CycleDuration}^{*}\right)}_{side,limb}^{trial}={\left(t_{{P2}^{*}}\right)}_{side,limb}^{trial}-{\left(t_{{P1}^{*}}\right)}_{side,limb}^{trial}$$

The “swimming” duty factor (abbreviated in the text as DutyFactor) is an analogy of the duty factor in terrestrial locomotion, and defined as the ratio of the duration of the propulsion phase to the swimming cycle duration [4]. It is estimated from the veterinarian’s data ${\left(DutyFactor\right)}_{side,limb}^{trial}$ (Equation (4)) and from the inertial sensor data ${\left({DutyFactor}^{*}\right)}_{side,limb}^{trial}$ (Equation (5)).

(4) $${\left(DutyFactor\right)}_{side,limb}^{trial}=100\frac{{\left(t_{R1}\right)}_{side,limb}^{trial}-{\left(t_{P1}\right)}_{side,limb}^{trial}}{{\left(CycleDuration\right)}_{side,limb}^{trial}}$$

(5) $${\left({DutyFactor}^{*}\right)}_{side,limb}^{trial}=100\frac{{\left(t_{{R1}^{*}}\right)}_{side,limb}^{trial}-{\left(t_{{P1}^{*}}\right)}_{side,limb}^{trial}}{{\left({CycleDuration}^{*}\right)}_{side,limb}^{trial}}$$

### 2.3. Statistical Analysis

First, the normality of the $\Delta t_{event}$, CycleDuration, DutyFactor, ${CycleDuration}^{*}$ and ${DutyFactor}^{*}$ was assessed using the Lilliefors test [16]. The Lilliefors test was performed using the “lillietest” function in Matlab R2025b (The MathWorks, Inc. Natick, MA, USA). This function returns a logical result of ‘true’ or ‘false’ regarding whether to accept or reject the null hypothesis that the data follows a normal distribution at the 5% significance level. 

Agreements between CycleDuration and ${CycleDuration}^{*},$ and, DutyFactor and ${DutyFactor}^{*}$ were evaluated in accordance with COSMIN recommendations and checklist guidelines [17], using intraclass correlation coefficient and Bland–Altman analysis to examine absolute agreement and potential systematic bias. In our case, the intraclass correlation coefficient used is a two-way random-effects single intraclass correlation coefficient for absolute agreement (ICC(2,1)) according to the guideline of Koo et al. [18]. Then, we could deduce a “poor” agreement if ICC values were less than 0.5, “moderate” agreement if ICC values were between 0.5 and 0.75, “good” agreement if ICC values were between 0.75 and 0.9, and “excellent” agreement if ICC values were greater than 0.90. For Bland–Altman analysis [19] (Bland Altman), prior the calculations of the bias and 95% limits of agreement (LoA), we also test assumptions of normality and homoscedasticity of the residuals according to the guideline of Haghayegh et al. [20]. Normality testing was performed with the Lilliefors test [16], and homoscedasticity was verified with the Breusch–Pagan test [21]. In the Bland–Altman plot, the x-axis shows the mean of the measurements obtained from the veterinarians’ observations and those obtained from the IMUs, whilst the y-axis shows the difference between the value obtained by the IMUs and the measurements made by the veterinarians. Thus, a positive value for the difference indicates that the measurement taken by the IMUs is higher than that obtained by the veterinarians, and conversely if the difference is negative.

All calculation was performed with Matlab R2025b (The MathWorks, Inc. Natick, MA, USA) with the Statistics and Machine Learning Toolbox (version 25.2).

## 3. Results

As we took measurements on six horses across two trials (RT and LT), we were able to analyse 22 measurements for the forelimbs (two are missing because the left limb of horses #2 and #4 were out of the field of view during the RT trial). For the hindlimbs, we have all 24 analysable measurements.

**Figure 4:**
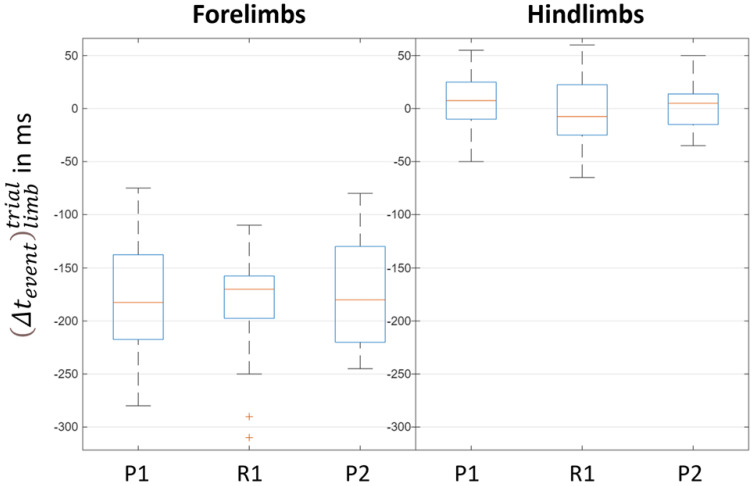
Box plot of timing differences ${\left(\Delta t_{event}\right)}_{limb}^{trial}$ between veterinarian-based and inertial sensor-based event detection for P1 (beginning of propulsion phase 1), R1 (beginning of retraction phase 1) and P2 (beginning of propulsion phase 2) for the six included horses. Missing data occurred when the veterinarian was unable to visualize the spatiotemporal event due to the left forelimb (LF) being out of the camera’s field of view. Thus, taking into account the six horses with two trials (RT and LT), for the limbs (RF, LF, RH) we have 12 measurements of events P1, R1 and P1. For the LF limb, we have only 10, as two measurements are missing.

All time differences ${\left(\Delta t_{event}\right)}_{limb}^{trial}$ are shown in Figure 4. A normality test was performed on each $\Delta t_{event}$ for the forelimbs (RF and LF) and the hindlimbs (RH and LH), demonstrating the “true” logical value of the Lilliefors test, which confirmed a normal distribution. Indeed, for the forelimbs, time differences were observed between the timing of the spatiotemporal events *P*1 (beginning of propulsion phase 1), *R*1 (beginning of the retraction phase 1) and *P*2 (beginning of propulsion phase 2) identified by the veterinarians and those estimated from the inertial sensors data *P*1*, *R*1* and *P*2* respectively. A mean time difference of −180 ms was noted, with a range from −310 ms to −70 ms. In contrast, for the hindlimbs, the correspondence was near-perfect, with a mean time difference of 10 ms and a range from −60 ms to 60 ms. 

The normality test on each CycleDuration deduced from the veterinarians’ data and ${CycleDuration}^{*}$ obtained from the inertial sensors demonstrated the “true” logical value of the Lilliefors test, which confirmed a normal distribution. 

**Figure 5:**
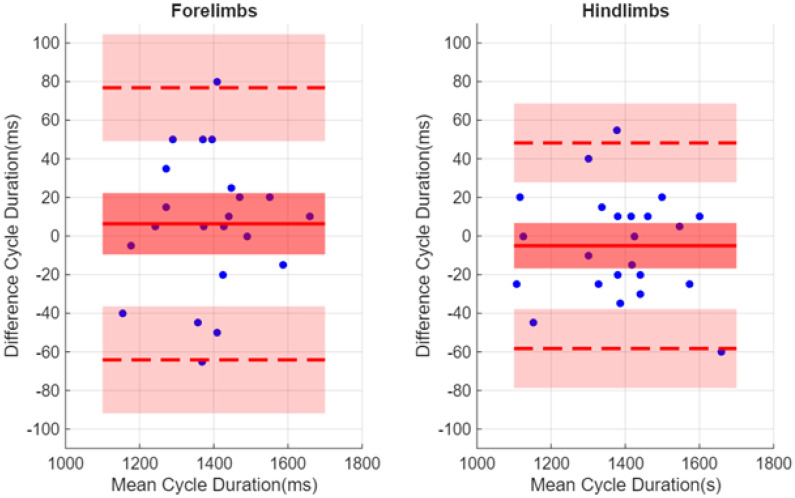
Bland–Altman plot comparing the CycleDuration deduced from the veterinarians’ data and ${CycleDuration}^{*}$ obtained from the inertial sensors. Solid colour red line denotes the bias and dashed lines upper and lower limits of agreement (LoA). Red and pink shaded areas indicate the 95% confidence interval (95% CI) of bias, lower and upper agreement limits. A total of 22 CycleDuration and ${CycleDuration}^{*}$ measurements were analysed for the forelimbs, as two measurements are missing for the LF limb, and 24 for the hindlimbs. A positive value for the difference indicates that the swim cycle duration obtained with the IMUs (${CycleDuration}^{*}$) is greater than that obtained by the veterinarians (CycleDuration) using the GoPros (GoPro Hero 8 Black, GoPro, San Mateo, CA, USA).

In the Bland–Alman plot comparing CycleDuration and ${CycleDuration}^{*}$, a similar residual dispersion was observed for both the forelimbs and hindlimbs (Figure 5). Specifically, a bias of 6 ms (95% CI: 20 ms, −10 ms) was found for the forelimbs and −5 ms (95% CI: 10 ms, −20 ms) for the hindlimbs. The limits of agreement (LoA) also showed slight differences, with upper LoA and lower LoA being respectively 80 ms (95% CI: 100 ms, 50 ms) and −60 ms (95% CI: −40 s, −90 ms) for the forelimbs, and 50 ms (95% CI: 70 ms, 30 ms) and −60 s (95% CI: −40 ms, −80 ms) for the hindlimbs. For absolute agreement, we found ICC(2,1) = 0.960 (95% CI: 0.904, 0.983) for the forelimbs and ICC(2,1) = 0.984 (95% CI: 0.963, 0.993) for the hindlimbs. These results demonstrated a “excellent” level of agreement for the estimation of the cycle duration between the both method and for the both limbs according the criteria.

The normality test on each “swimming” duty factor, DutyFactor, deduced from the veterinarians’ data and ${DutyFactor}^{*}$ obtained from the inertial sensors demonstrated the “true” logical value of the Lilliefors test, which confirmed a normal distribution.

**Figure 6:**
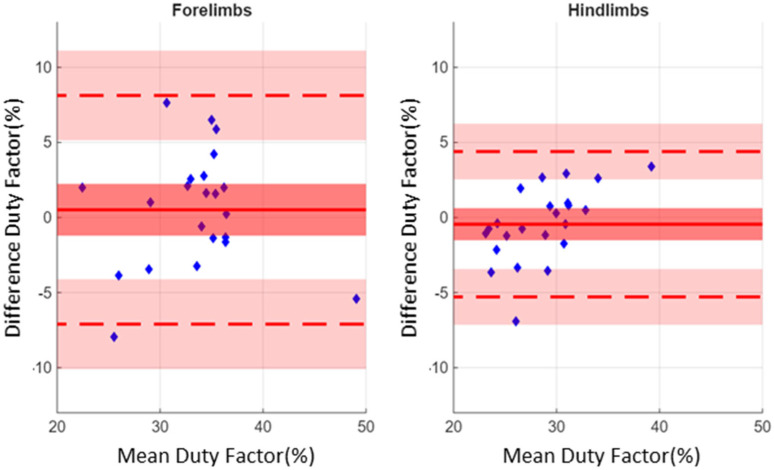
Bland–Altman plot comparing the “swimming” dutyfactor (DutyFactor) deduced from the veterinarians’ data and ${DutyFactor}^{*}$ obtained from the inertial sensors. Solid colour red line denotes the bias, and dashed lines upper and lower limits of agreement (LoA). Red and pink shaded areas indicate the 95% confidence interval (95% CI) of bias, lower and upper agreement limits. A total of 22 DutyFactor and ${DutyFactor}^{*}$ measurements were analysed for the forelimbs, as two measurements are missing for the LF limb, and 24 for the hindlimbs. A positive value for the difference indicates that the ${DutyFactor}^{*}$ obtained with the IMUs is greater than DutyFactor obtained by the veterinarians using the GoPros.

Small bias values were observed in the Bland–Alman plot comparing DutyFactor and ${DutyFactor}^{*}$ (Figure 6) with a bias of 0.51% (95% CI: 2.24%, −1.21%) for the forelimbs, and −0.44% (95% CI: 0.62%, −1.15%) for the hindlimbs (Figure 6). However, the limits of agreement (LoA) were relatively larger for the forelimbs. We noticed upper and lower LoA of 8.12% (95% CI: 11.12%, 5.14%) and −7.10% (95% CI: −4.11%, −10.08%) for the forelimbs, and 4.39% (95% CI: 6.21%, 2.53%) and −5.28% (95% CI: −3.42%, −7.14%) for the hindlimbs, respectively. For absolute agreement, we found ICC(2,1) = 0.766 (95% CI: 0.508, 0.893) for the forelimbs, and ICC(2,1) = 0.823 (95% CI: 0.625, 0.920) for hindlimbs. 

## 4. Discussion

The present study revealed time differences between the spatiotemporal events as estimated by veterinarians through visual observations and those determined by inertial sensors for the forelimbs during swimming sessions. In contrast, a greater degree of temporal consistency was observed between the two methods for the hindlimbs. However, an “excellent” absolute agreement was found between the two methods according to ICC analysis, with cycle duration values being highly similar for both forelimbs and hindlimbs. However, when assessing the agreement between the two methods, we observed, for the estimation of the DutyFactor for the forelimbs, a limit of agreement ranging from 11.12% to −10.08% in the worst-case scenario (which takes into account the 95% CI) despite an absolute agreement considered to be “moderate” to “excellent”.

The time difference observed in the timing determinations of propulsion and return phases between visual observations by veterinarians and measurements obtained from inertial sensors for the forelimbs, as well as the timing consistency observed for the hindlimbs, can be explained by the specific anatomical architecture of the horse’s limbs. Veterinarians identified propulsion and return motions by observing the proximal part of the limbs, such as the rotation of the elbow joint for the forelimbs and the rotation of the stifle joint for the hindlimbs. In contrast, the inertial sensors, located on the metacarpus region, reflected the motion of the distal part of the limbs, capturing rotations primarily at the carpal joint for the forelimb and the tarsal joint for the hindlimb. The horse’s hindlimbs have a unique anatomical peculiarity known as the reciprocal apparatus [7]. It is a tendinous system (the tendinous peroneus tertius muscle and the tendon of the superficial digital flexor muscle) on either side of the tibia that allows passive support of the limb with minimal muscular effort [22]. This apparatus mechanically couples the flexion and extension movements of the stifle and tarsal joints [7], a phenomenon observable in kinematic analyses [22,23]. Specifically, during both terrestrial locomotion [7] and swimming [9], the flexion–extension angles of the elbow and carpal joints show a phase difference, whereas the flexion–extension angles of the stifle and tarsal joints remain in phase. As a result, during swimming, inertial sensors placed on the metatarsal region provide a consistent proxy for stifle joint motion in the hindlimbs, leading to coherent timing of spatiotemporal events between the two methods. However, it is not recommended for the purpose of assessing forelimb–hindlimb coordination, as the detected spatiotemporal events for the forelimbs and hindlimbs are not directly comparable. 

We observed an “excellent” absolute agreement in determining the swimming cycle durations (CycleDuration) between the visual observations made by veterinarians and the estimations obtained from inertial sensors for both forelimbs and hindlimbs. At first glance, this observation may seem to contradict the findings discussed in the previous paragraph. However, in the context of rhythmic motion, cycle duration primarily depends on the consistent identification of the same intercycle features [24]. In our study, although the time determination of zero values on metacarpal/metatarsal ${\omega z}_{norm}$ does not correspond to the same spatiotemporal events for the proximal part of the limb for forelimbs and hindlimbs as discussed in the previous paragraph, the rhythmic nature of swimming allowed these features to be used reliably for determining the cycle duration of each individual limb. Moreover, the Bland–Altman analysis enables the determination of limits of agreement (which take into account the 95% confidence interval) ranging from 100 ms to −90 ms for the forelimbs and from 70 ms to −80 ms for the hindlimbs. These limits therefore define the boundaries of the method used here and may serve as an objective benchmark for comparison with other methods [25], or for the evaluation of the Minimal Detectable Change (MDC) of this method [20]. This latter aspect will be important in the context of future studies, such as interventional studies during rehabilitation.

The results regarding “swimming” duty factor (DutyFactor) show contrasting outcomes. For the hindlimbs, based on the consistency in the timing estimations of spatiotemporal events by both veterinarians and inertial sensors (Figure 4), a very low bias was observed (Figure 5), which supports the good average accuracy of inertial sensor-based estimations in the six horses evaluated in the present study. Conversely, for the forelimbs, due to the mismatch between the spatiotemporal events determined by the inertial sensors and those defined through visual observation by veterinarians, the ratios calculated from inertial sensor data directly correspond to those defined by Equation 4. However, Figure 4 demonstrates a consistency in the time offsets between the two methods for the forelimbs, which explains why the bias observed in Figure 5 was comparable to that found for the hindlimbs. Nevertheless, for both the forelimbs and hindlimbs, wide limits of agreement were observed. The Bland–Altman analysis enables the determination of limits of agreement (which take into account the 95% confidence interval) ranging from 6.21% to −7.14% for the hindlimbs, and ranging from 11.12% to −10.08% for the forelimbs. These large ranges can be attributed to the fact that the duty factor is a ratio and, as such, is highly sensitive to error propagation [26]. These observations are confirmed by a “moderate” absolute agreement noticed between the two methods. Consequently, we recommend here interpreting “swimming” duty factor (DutyFactor) estimations with caution.

Our study has limitations. First, the number of horses investigated was limited. To mitigate this, two trials were conducted for each horse. Indeed, right- and left-hand trials differ fundamentally in that, in the case of the left-hand trial, the horse enters the measurement zone after completing a turn, whereas in the case of the right-hand trial, the horse enters the measurement zone after swimming along a straight section (Figure 1). These constitute two distinct conditions for the horse, as it is potentially likely to alter its swimming strategy [4]. Second, the veterinarians’ observations are inherently subject to a degree of subjectivity. However, a previous study [4] found that the three veterinarians identified the events within a range of two frames when the video was recorded at 120 Hz. Therefore, we could hypothesize that their detections had a precision of 20 ms [4]. This was considered to be our precision threshold [27]. This precision was smaller than the differences observed between the two methods, suggesting that observer variability did not influence the overall results.

## 5. Conclusions

We evaluated the agreement of using inertial sensors placed on the metacarpal/metatarsal regions to determine spatiotemporal events for the forelimbs and hindlimbs during horse swimming. Our results suggest that the spatiotemporal events estimated by the hindlimb inertial sensors were the most reliable for studying horse spatiotemporal parameters during swimming. Indeed, for the hindlimb, an “excellent” agreement has been demonstrated for the determination of swim cycle duration (ICC = 0.96, 95% CI: 0.904–0.983), and “swimming” duty factor (DutyFactor) demonstrated “moderate” to “excellent” agreement (ICC = 0.82; 95% CI: 0.625–0.920). However, the wide confidence intervals obtained for the “swimming” duty factor suggest caution should be taken when using this parameter, obtained via IMUs as configured in this study, to assess changes associated with rehabilitation regimens. This study was a necessary step towards the use of IMUs for monitoring a horse’s swimming parameters. By establishing the level and limits of agreement for the spatiotemporal parameters of swimming using IMUs, we will be able to assess the practical limitations of using IMUs in interventional studies during rehabilitation.

## Data Availability

The original contributions presented in this study are included in the article. Further inquiries can be directed to the corresponding author.

## Abbreviations

The following abbreviations are used in this manuscript:

ms	Milliseconds
ANOVA	Analysis of Variance
ICC	Intraclass Correlation Coefficient
LoA	Limits of Agreement
95% CI	95% confidence interval

## References

[1] Muñoz A., Becero M., Saitua A., Argüelles D., De Medina A.S., Castejón-Riber C. (2019). Exercise in the Water: Should We Incorporate It into Training and Rehabilitation Programs for the Sport Horse?. Atti Della Accad. Peloritana Dei Pericolanti-Cl. Di Sci. Med.-Biol..

[2] Steel C., Bond B., Morrice-West A. (2019). Survey of Trainers on the Use of Swimming Exercise for Standardbred Racehorses in Australia. Aust. Vet. J..

[3] Imahara T. (1976). Swimming Pool for Horses. Exp. Rep. Equine Health Lab..

[4] Gaulmin P., Marin F., Moiroud C., Beaumont A., Jacquet S., De Azevedo E., Martin P., Audigié F., Chateau H., Giraudet C. (2025). Description and Analysis of Horse Swimming Strategies in a U-Shaped Pool. Animals.

[5] Toussain H., Hollande A.P., Berg C., Vorontsov A. (2000). Biomechanics of Swimming. Exercise and Sport Science.

[6] Fish F.E. (1996). Transitions from Drag-Based to Lift-Based Propulsion in Mammalian Swimming. Am. Zool..

[7] Back W., Clayton H.M. (2013). Equine Locomotion.

[8] Denoix J.-M. (2019). Essentials in Clinical Anatomy of the Equine Locomotor System.

[9] Giraudet C., Moiroud C., Beaumont A., Gaulmin P., Hatrisse C., Azevedo E., Denoix J.-M., Ben Mansour K., Martin P., Audigié F. (2023). Development of a Methodology for Low-Cost 3D Underwater Motion Capture: Application to the Biomechanics of Horse Swimming. Sensors.

[10] Santosuosso E., Leguillette R., Vinardell T., Filho S., Massie S., McCrae P., Johnson S., Rolian C., David F. (2021). Kinematic Analysis During Straight Line Free Swimming in Horses: Part 1-Forelimbs. Front. Vet. Sci..

[11] Marin F. (2020). Human and Animal Motion Tracking Using Inertial Sensors. Sensors.

[12] Crecan C.M., Peștean C.P. (2023). Inertial Sensor Technologies—Their Role in Equine Gait Analysis, a Review. Sensors.

[13] Fercher C., Bartsch J., Kluge S., Schneider F., Liedtke A.M., Schleichardt A., Ueberschär O. (2024). Applying Multi-Purpose Commercial Inertial Sensors for Monitoring Equine Locomotion in Equestrian Training. Sensors.

[14] Sapone M., Martin P., Ben Mansour K., Chateau H., Marin F. (2021). The Protraction and Retraction Angles of Horse Limbs: An Estimation during Trotting Using Inertial Sensors. Sensors.

[15] Ekdahl M., Loewen A., Erdman A., Sahin S., Ulman S. (2023). Inertial Measurement Unit Sensor-to-Segment Calibration Comparison for Sport-Specific Motion Analysis. Sensors.

[16] Lilliefors H.W. (1967). On the Kolmogorov-Smirnov Test for Normality with Mean and Variance Unknown. J. Am. Stat. Assoc..

[17] Mokkink L.B., De Vet H.C.W., Prinsen C.A.C., Patrick D.L., Alonso J., Bouter L.M., Terwee C.B. (2018). COSMIN Risk of Bias Checklist for Systematic Reviews of Patient-Reported Outcome Measures. Qual. Life Res..

[18] Koo T.K., Li M.Y. (2016). A Guideline of Selecting and Reporting Intraclass Correlation Coefficients for Reliability Research. J. Chiropr. Med..

[19] Martin Bland J., Altman D.G. (1986). Statistical Methods for Assessing Agreement between Two Methods of Mlinical Measurement. Lancet.

[20] Haghayegh S., Kang H.-A., Khoshnevis S., Smolensky M.H., Diller K.R. (2020). A Comprehensive Guideline for Bland–Altman and Intra Class Correlation Calculations to Properly Compare Two Methods of Measurement and Interpret Findings. Physiol. Meas..

[21] Breusch T.S., Pagan A.R. (1979). A Simple Test for Heteroscedasticity and Random Coefficient Variation. Econometrica.

[22] Van Weeren P.R., Van Den Bogert A.J., Barneveld A., Hartman W., Kersjes A.W. (1990). The Role of the Reciprocal Apparatus in the Hind Limb of the Horse Investigated by a Modified CODA-3 Opto-electronic Kinematic Analysis System. Equine Vet. J..

[23] Van Weeren P.R., Jansen M.O., Van Den Bogert A.J., Barneveld A. (1992). A Kinematic and Strain Gauge Study of the Reciprocal Apparatus in the Equine Hind Limb. J. Biomech..

[24] Robertson D.G.E., Caldwell G.E., Hamill J., Kamen G., Whittlesey S.N. (2014). Research Methods in Biomechanics.

[25] Montenij L.J., Buhre W.F., Jansen J.R., Kruitwagen C.L., De Waal E.E. (2016). Methodology of Method Comparison Studies Evaluating the Validity of Cardiac Output Monitors: A Stepwise Approach and Checklist. Br. J. Anaesth..

[26] Gertsbakh I. (2003). Measurement Theory for Engineers.

[27] Menditto A., Patriarca M., Magnusson B. (2007). Understanding the Meaning of Accuracy, Trueness and Precision. Accredit. Qual. Assur..

